# Covid-19 risk data during lockdown-like policy in Indonesia

**DOI:** 10.1016/j.dib.2021.106801

**Published:** 2021-01-28

**Authors:** Khreshna Syuhada, Aqilah Wibisono, Arief Hakim, Fida Addini

**Affiliations:** Statistics Research Division, Institut Teknologi Bandung, 40132, Indonesia

**Keywords:** Covid-19 pandemic, Risk data, Risk measure, Stochastic forecast, Value-at-risk

## Abstract

Covid-19 pandemic has spread fast almost all countries in the world including Indonesia. In order to slow such pandemic confirmed cases, Indonesian local and central governments apply a lockdown-like policy. We call this Large-Scale Social Restriction (Pembatasan Sosial Berskala Besar, known as PSBB) and PSBB-variant that is Expanded and Tightened Social Restriction or Pembatasan Sosial yang Diperluas dan Diperketat (PSDD). In this paper, we present number of cases and case fatality rate before, during and after such lockdown-like policy. This article contains Covid-19 risk data of several cities and provinces in Indonesia. We have used central and local government Covid-19 tracking sites to determine the daily risks for several cities and provinces in Indonesia. All data were extracted on August 22, 2020. We developed these data and calculated daily rate of confirmed and active cases, case fatality rate and rate of case fatality rate before, during and after lockdown-like policy. Furthermore, such risk modeling is used to forecast of what so-called Value-at-Risk (VaR).

## Specifications Table

**Table:** 

Subject	Public Health and Health Policy
Specific subject area	Health Risk and Health Forecast
Type of data	Table Figure Raw data
How data were acquired	By using central and local government Covid-19 tracking sites, we extracted the number of confirmed Covid-19 cases, the number of active cases and the number of deaths. We then calculated daily rate of confirmed and active cases, case fatality rate and rate of case fatality rate. All data were extracted on August 22, 2020.
Data format	Raw Analyzed Excel file of the daily data are presented in Supplementary Data. The data were analyzed using the statistical software of R.
Parameters for data collection	Provinces and cities included in these data are located in major islands in Indonesia. In addition, provinces included in these data are among the top ten provinces with most confirmed Covid-19 cases in Indonesia. Cities included in these data are cities with daily data available on the local government Covid-19 tracking site.
Description of data collection	We developed these data and calculated daily rate of confirmed cases, rate of active cases, case fatality rate and rate of case fatality rate. The Covid-19 risks are grouped according to lockdown-like policy.
Data source location	Country: Indonesia Raw data can be retrieved from https://covid19.go.id/peta-sebaran, https://pikobar.jabarprov.go.id, https://covid19.papua.go.id, https://covid19.pemkomedan.go.id, https://covid19.kaltimprov.go.id.
Data accessibility	Summary data are available with this article. Raw data including R code used for analysis are available in Mendeley Data (https://data.mendeley.com/datasets/stksys9c3r/1).

## Value of the Data

• Daily Covid-19 risk data of confirmed cases, active cases and case fatality rate are presented during lockdown-like policy to provide information related to the increase or decrease of the risk, i.e., to carry out a risk modeling.
• These data may be used by policy makers to make decision whether to continue lockdown-like policy or not and to forecast future cases.
• The Covid-19 risk data may increase people awareness in making social or physical distancing to slow pandemic spread.

## 1 Data Description

The daily prevalence data of Covid-19 to August 22, 2020 were collected from central and local government Covid-19 tracking site (https://covid19.go.id/peta-sebaran, https://pikobar.jabarprov.go.id, https://covid19.papua.go.id, https://covid19.pemkomedan.go.id, https://covid19.kaltimprov.go.id) [1], [2], [3], [4], [5]. The starting dates for each city and province were as follows: City of Bandung: March 6, 2020, Province of West Java: March 2, 2020, Province of South Sulawesi: March 19, 2020, City of Jayapura: April 3, 2020, Province of Papua: March 22, 2020, City of Medan: March 27, 2020, Province of North Sumatera: March 17, 2020, City of Samarinda: April 4, 2020, and Province of East Kalimantan: March 14, 2020. It was known that the governments in the first three provinces and the corresponding capital cities applied lockdown-like policy called PSBB (Pembatasan Sosial Berskala Besar) or PSDD (Pembatasan Sosial yang Diperluas dan Diperketat). PSBB and PSDD are actually similar. The Health Minister regulated the former and several local governments then implemented it under his permission [6]. Meanwhile, the latter had been regulated only by the local government of Province of Papua [7] before the regulation of PSBB was set up by the Health Minister. The dates of these lockdown-like policies were 6 May to 25 June (PSBB in Bandung/West Java), 24 April to 22 May (PSBB in South Sulawesi), 24 April to 22 August (PSDD in Jayapura), 24 April to 3 August (PSDD in Papua). The data for such three provinces and their capital cities were grouped according to the period of pre-, during and post-lockdown-like policy, where the period of pre-lockdown-like policy was defined from the above starting date to one day before lockdown-like policy. Meanwhile, the corresponding period of post-lockdown-like policy was from one day after lockdown-like policy to August 22, 2020. Note that the latter period was not defined in City of Jayapura since PSDD still applied in this city on August 22, 2020. Note also that, unlike the other three provinces, North Sumatera and East Kalimantan experienced the period of pre-lockdown-like policy only since they did not enforce the lockdown-like policy. The obtained data were then used to carry out a risk modeling based on daily rates and thus to forecast of what so-called Value-at-Risk (VaR).

We define several risks as follows. The first risk, called confirmed cases rate $\left(R_{t}\right)$, is a natural logarithm of number of confirmed cases $\left(C_{t}\right)$ up to time t over the one up to time t−1, $R_{t}=\ln\left(C_{t}/C_{t-1}\right)$. The case fatality rate at time t, called $CFR_{t}$, is defined as (total) number of deaths up to time t, $D_{t}$, over (total) number of confirmed cases up to time t given by $CFR_{t}=D_{t}/C_{t}$ [8]. The active cases rate $\left(RA_{t}\right)$ is a natural logarithm of number of active cases $\left(A_{t}\right)$ up to time t over the one up to time t−1, $RA_{t}=\ln\left(A_{t}/A_{t-1}\right)$, where the active cases provide information on number of confirmed cases subtracted by number of deaths and number of recoveries. The last risk is rate of case fatality rate $\left(RCFR_{t}\right)$ defined as a natural logarithm of case fatality rate $\left(CFR_{t}\right)$ at time t over the one up to time t−1, $RCFR_{t}=\ln\left(CFR_{t}/CFR_{t-1}\right)$.

**Fig. 1:**
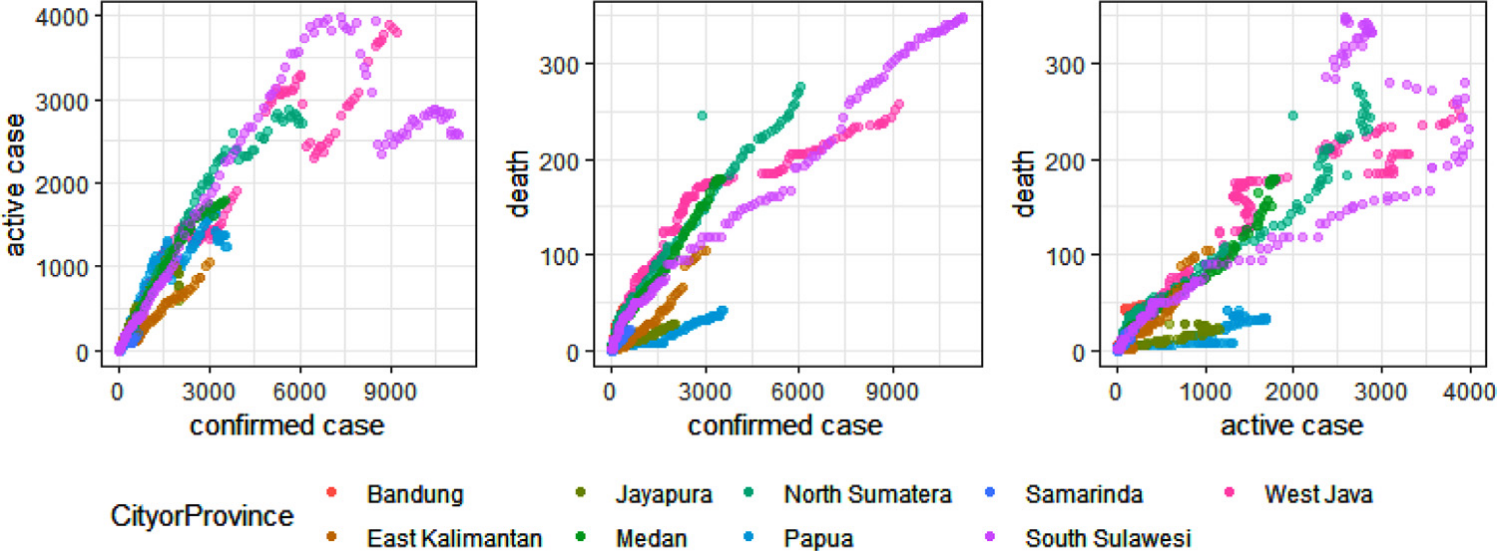
Scatter plots of number of confirmed cases against number of active cases (and number of deaths) and number of active cases against number of deaths for cities and provinces in Indonesia.

**Fig. 2:**
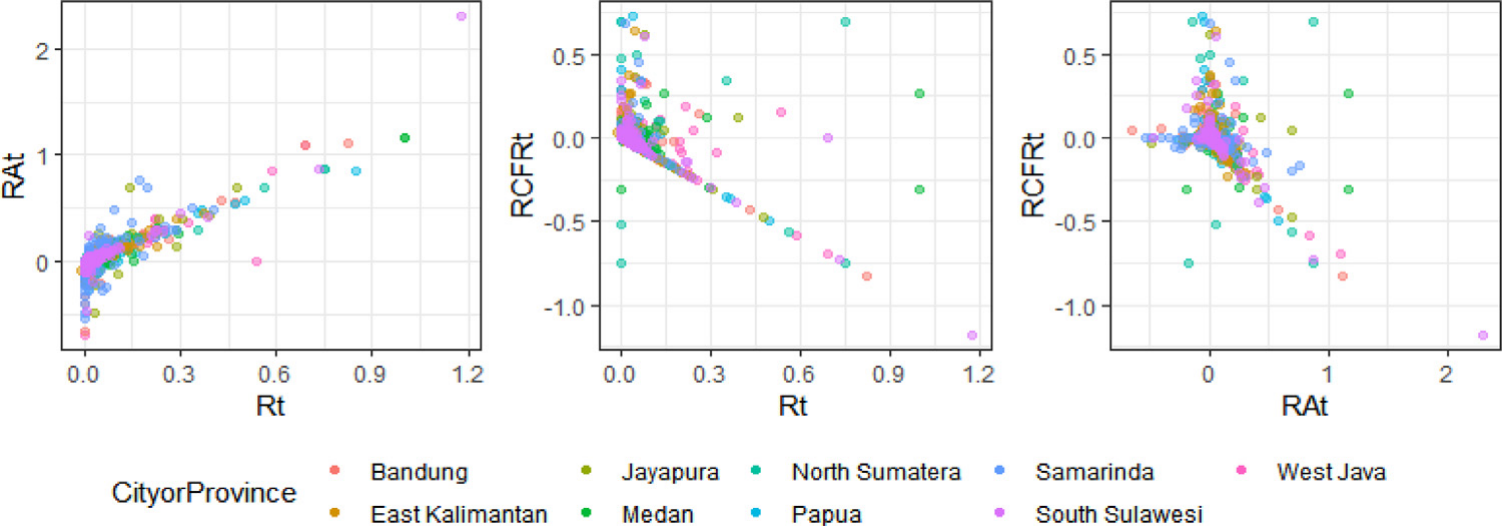
Scatter plots of confirmed cases rate against active cases rate (and rate of case fatality rate) and active cases rate against rate of case fatality rate for cities and provinces in Indonesia.

Number of confirmed cases, number of active cases and number of deaths defining the above risks are plotted in Fig. 1. Their plots show that number of confirmed cases and number of active cases are positively and strongly correlated which means that the increase of the former is followed by that of the latter. Furthermore, they are also correlated to number of deaths. The resulting risks are then displayed in Fig. 2.

Table 1, Table 2, Table 3 summarize statistics of risks of confirmed cases rate, active case rate, case fatality rate and rate of case fatality rate in three provinces (West Java, South Sulawesi and Papua) along with their capital cities in the period of before, during and after lockdown-like policy. Meanwhile, statistics of such risks for the other provinces (North Sumatera and East Kalimantan) along with their capital cities are summarized in Table 4. Raw data and R code used for this analysis are available at https://data.mendeley.com/datasets/stksys9c3r/1.

**Table 1:** Statistics of risks in the period of pre-, during and post-lockdown-like policy (PSBB) in City of Bandung and Province of West Java.

	$R_{t}$				$CFR_{t}$		
	Pre-PSBB	PSBB	Post-PSBB		Pre-PSBB	PSBB	Post-PSBB
**Bandung**				**Bandung**			
Mean	0.1089	0.0137	0.0140	Mean	0.1200	0.1256	0.0920
Q1	0.0000	0.0000	0.0020	Q1	0.0000	0.1140	0.0757
Median	0.0000	0.0000	0.0059	Median	0.1500	0.1209	0.0986
Q3	0.1329	0.0086	0.0277	Q3	0.2000	0.1339	0.1096
Min	0.0000	0.0000	0.0000	Min	0.0000	0.1049	0.0588
Max	0.8267	0.1711	0.0775	Max	0.2500	0.1733	0.1117
**West Java**				**West Java**			
Mean	0.1008	0.0167	0.0195	Mean	0.1345	0.0639	0.0368
Q1	0.0133	0.0076	0.0098	Q1	0.0904	0.0619	0.0305
Median	0.0508	0.0134	0.0142	Median	0.1081	0.0635	0.0345
Q3	0.1367	0.0192	0.0213	Q3	0.1429	0.0657	0.0374
Min	0.0000	0.0000	0.0044	Min	0.0000	0.0581	0.0271
Max	0.6931	0.0987	0.2219	Max	0.5000	0.0734	0.0581

	$RA_{t}$				$RCFR_{t}$		
	Pre-PSBB	PSBB	Post-PSBB		Pre-PSBB	PSBB	Post-PSBB

**Bandung**				**Bandung**			
Mean	0.1087	0.0088	0.0059	Mean	-0.0122	0.1256	-0.0100
Q1	0.0000	0.0000	0.0000	Q1	-0.0269	-0.0078	-0.0150
Median	0.0000	0.0000	0.0161	Median	0.0000	0.0000	-0.0053
Q3	0.1484	0.0120	0.0389	Q3	0.0692	0.0000	0.0000
Min	-0.3365	-0.4078	-0.6568	Min	-0.8267	-0.1768	-0.0775
Max	1.1137	0.2398	0.2079	Max	0.3254	0.1419	0.0473
**West Java**				**West Java**			
Mean	0.0962	0.0061	0.0171	Mean	-0.0306	-0.0032	-0.0126
Q1	0.0000	-0.0119	0.0033	Q1	-0.0621	-0.0134	-0.0155
Median	0.0428	0.0052	0.0150	Median	-0.0094	-0.0066	-0.0093
Q3	0.1151	0.0174	0.0274	Q3	0.0082	0.0037	-0.0045
Min	-0.6931	-0.0532	-0.1861	Min	-0.6931	-0.0987	-0.2002
Max	1.0986	0.1406	0.3971	Max	0.3494	0.0955	0.0221

**Table 2:** Statistics of risks in the period of pre-, during and post-lockdown-like policy (PSBB) in Province of South Sulawesi.

	$R_{t}$				$CFR_{t}$		
	Pre-PSBB	PSBB	Post-PSBB		Pre-PSBB	PSBB	Post-PSBB
Mean	0.1512	0.0383	0.0243	Mean	0.1595	0.0652	0.0360
Q1	0.0081	0.0261	0.0117	Q1	0.0854	0.0574	0.0317
Median	0.0465	0.0383	0.0221	Median	0.0909	0.0658	0.0334
Q3	0.2131	0.0529	0.0325	Q3	0.1091	0.0693	0.0384
Min	0.0000	0.0000	0.0023	Min	0.0426	0.0486	0.0294
Max	1.1787	0.1080	0.0841	Max	0.5000	0.0818	0.0515

	$RA_{t}$				$RCFR_{t}$		
	Pre-PSBB	PSBB	Post-PSBB		Pre-PSBB	PSBB	Post-PSBB

Mean	0.1821	0.0326	0.0138	Mean	-0.0504	-0.0193	-0.0050
Q1	0.0029	0.0108	-0.0052	Q1	-0.1208	-0.0386	-0.0220
Median	0.0445	0.0406	0.0141	Median	-0.0002	-0.0263	-0.0064
Q3	0.2600	0.0645	0.0396	Q3	0.0455	0.0000	0.0056
Min	-0.1989	-0.1081	-0.4705	Min	-1.1787	-0.1080	-0.0700
Max	2.3026	0.1218	0.2475	Max	0.6115	0.0389	0.1366

**Table 3:** Statistics of risks in the period of pre-, during and post-lockdown-like policy (PSDD) in City of Jayapura and Province of Papua.

	$R_{t}$				$CFR_{t}$		
	Pre-PSDD	PSDD	Post-PSDD		Pre-PSDD	PSDD	Post-PSDD
**Jayapura**				**Jayapura**			
Mean	0.0755	0.0379		Mean	0.1059	0.0229	
Q1	0.0000	0.0013		Q1	0.0952	0.0130	
Median	0.0418	0.0170		Median	0.1036	0.0139	
Q3	0.0786	0.0418		Q3	0.1278	0.0224	
Min	0.0000	0.0000		Min	0.0000	0.0103	
Max	0.4796	0.3909		Max	0.1538	0.0789	
**Papua**				**Papua**			
Mean	0.1178	0.0311	0.0071	Mean	0.0432	0.0129	0.0108
Q1	0.0000	0.0108	0.0028	Q1	0.0000	0.0080	0.0105
Median	0.0484	0.0203	0.0061	Median	0.0544	0.0104	0.0106
Q3	0.1094	0.0402	0.0093	Q3	0.0648	0.0153	0.0108
Min	0.0000	0.0000	0.0000	Min	0.0000	0.0042	0.0104
Max	0.8473	0.1589	0.0228	Max	0.1154	0.0441	0.0119

	$RA_{t}$				$RCFR_{t}$		
	Pre-PSDD	PSDD	Post-PSDD		Pre-PSDD	PSDD	Post-PSDD

**Jayapura**				**Jayapura**			
Mean	0.0385	0.0339		Mean	0.0018	-0.0170	
Q1	-0.0455	-0.0018		Q1	-0.0703	-0.0343	
Median	0.0000	0.0128		Median	-0.0323	-0.0099	
Q3	0.0963	0.0508		Q3	0.0000	0.0000	
Min	-0.2231	-0.4884		Min	-0.4796	-0.3075	
Max	0.6931	0.6931		Max	0.6131	0.1447	
**Papua**				**Papua**			
Mean	0.1070	0.0285	-0.0158	Mean	-0.0152	-0.0144	0.0055
Q1	0.0000	0.0055	-0.0235	Q1	-0.1094	-0.0368	-0.0074
Median	0.0000	0.0264	-0.0007	Median	-0.0603	-0.0169	-0.0029
Q3	0.1248	0.0526	0.0044	Q3	0.0000	-0.0066	0.0027
Min	-0.1754	-0.2677	-0.1309	Min	-0.5000	-0.1589	-0.0159
Max	0.8473	0.1705	0.0418	Max	0.6931	0.7229	0.0809

**Table 4:** Statistics of risks in City of Medan, Province of North Sumatera, City of Samarinda and Province of East Kalimantan.

	$R_{t}$	$RA_{t}$	$CFR_{t}$	$RCFR_{t}$		$R_{t}$	$RA_{t}$	$CFR_{t}$	$RCFR_{t}$
**Medan**					**Samarinda**				
Mean	0.0430	0.0397	0.0810	-0.0069	Mean	0.0381	0.0299	0.0213	0.0010
Q1	0.0060	0.0000	0.0527	-0.0196	Q1	0.0000	0.0000	0.0000	-0.0301
Median	0.0230	0.0133	0.0753	0.0000	Median	0.0037	0.0000	0.0233	0.0000
Q3	0.0523	0.0537	0.1024	0.0088	Q3	0.0502	0.0978	0.0342	0.0000
Min	0.0000	-0.1989	0.0489	-0.3054	Min	0.0000	-0.5390	0.0000	-0.1975
Max	0.9985	1.1632	0.1875	0.2589	Max	0.4055	0.7621	0.0575	0.6790
**North Sumatera**					**East Kalimantan**				
Mean	0.0420	0.0377	0.0940	-0.0064	Mean	0.0386	0.0346	0.0168	-0.0029
Q1	0.0071	0.0000	0.0525	-0.0205	Q1	0.0083	-0.0020	0.0101	-0.0341
Median	0.0203	0.0152	0.0884	-0.0027	Median	0.0236	0.0139	0.0133	-0.0130
Q3	0.0523	0.0610	0.1230	0.0037	Q3	0.0460	0.0751	0.0231	0.0000
Min	0.0000	-0.1744	0.0432	-0.7492	Min	-0.0109	-0.2048	0.0000	-0.2288
Max	0.7492	0.8675	0.2500	0.6931	Max	0.3747	0.4418	0.0526	0.6425

## 2 Experimental Design, Materials and Methods

**Fig. 3:**
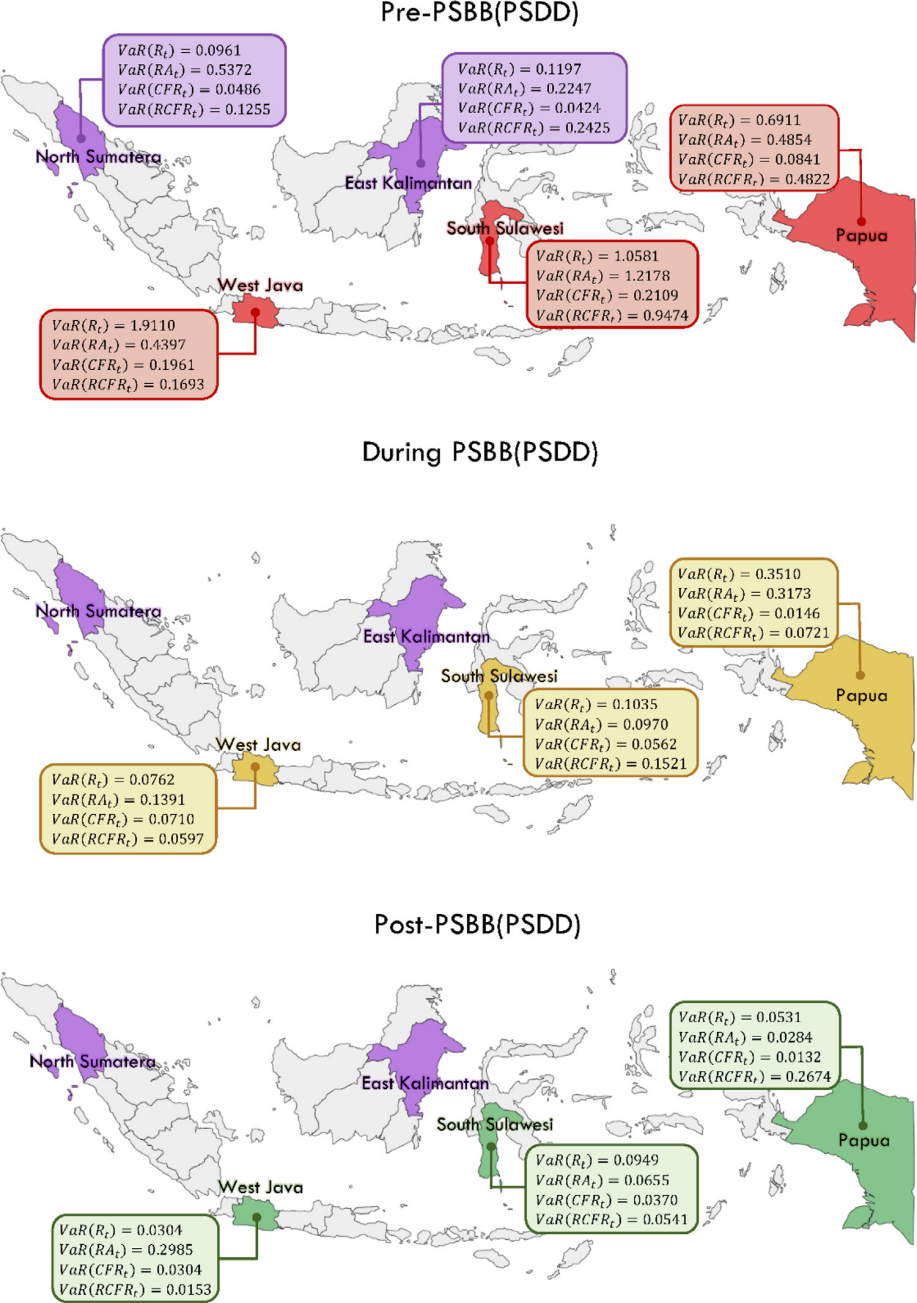
Value-at-Risk forecast of Covid-19 risks with the best accuracy in the period of pre-, during and post-lockdown-like policy in all provinces according to the map of Indonesia.

Using the local government sites, we extracted risk data of confirmed cases rate, active cases rate, case fatality rate and rate of case fatality rate. The data were collected before, during and after lockdown-like policy, i.e., pre-PSBB/PSDD, PSBB/PSDD, and post-PSBB/PSDD. We analyzed such daily data and constructed two stochastic models: one model with constant volatility and the other model known as heteroscedastic model (model with dynamic volatility). The former was called quantile autoregressive (QAR) model whilst the latter was quantile autoregressive conditional heteroscedastic (QARCH) model [9], [10], [11]. Covid-19 risk forecasting is conducted by using the risk measure concept commonly used in finance and insurance. In particular, we forecast future confirmed cases rate, active cases rate, case fatality rate and rate of case fatality rate by a what so-called Value-at-Risk (VaR). It is a maximum risk that can be tolerated at a certain level of confidence. Table 5, Table 6, Table 7 display risk modeling of VaR forecast through the models of QAR and QARCH for West Java, South Sulawesi, Papua and their capital cities. Meanwhile, the VaR forecasts for North Sumatera, East Kalimantan and their capital cities are presented in Table 8. The accuracy of each VaR forecast is also provided in these tables in terms of coverage probability as in [12]. The VaR forecast obtained from the model with coverage probability whose value is closer to the 0.95 level of confidence shows the best accuracy and is presented in boldface. The best VaR forecasts for all regions are provided in Fig. 3 that represents the map of Indonesia. The forecasts for North Sumatera and East Kalimantan are provided in the period of pre-lockdown-like policy only since these provinces did not apply the lockdown-like policy as already stated before in the section of Data Description.

**Table 5:** Value-at-Risk forecast of Covid-19 risks in the period of pre-, during and post-lockdown-like policy (PSBB) in City of Bandung and Province of West Java at 0.95 level of confidence and the corresponding coverage probability (in parentheses).

	$R_{t}$				$CFR_{t}$		
	Pre-PSBB	PSBB	Post-PSBB		Pre-PSBB	PSBB	Post-PSBB
**Bandung**				**Bandung**			
QAR	**0.6910**	**0.0784**	**0.0401**	QAR	**0.2353**	0.1121	0.0605
	(0.9348)	(0.9231)	(0.9138)		(0.9787)	(0.1692)	(0.0517)
QARCH	1.8297	0.2718	0.1059	QARCH	0.4210	**0.1367**	**0.0671**
	(1.0000)	(1.0000)	(1.0000)		(1.0000)	(0.7692)	(0.1552)
**West Java**				**West Java**			
QAR	0.1467	0.0306	**0.0304**	QAR	0.0825	0.0627	0.0285
	(0.7538)	(0.8824)	(0.9310)		(0.1692)	(0.3725)	(0.1552)
QARCH	**1.9110**	**0.0762**	0.0721	QARCH	**0.1961**	**0.0710**	**0.0304**
	(1.0000)	(0.9804)	(0.9828		(0.8923)	(0.9608)	(0.2586)

	$RA_{t}$				$RCFR_{t}$		
	Pre-PSBB	PSBB	Post-PSBB		Pre-PSBB	PSBB	Post-PSBB

**Bandung**				**Bandung**			
QAR	**1.1546**	**0.0782**	0.0397	QAR	0.0731	**0.0466**	**0.0045**
	(1.0000)	(0.9231)	(0.7414)		(0.7333)	(0.9385)	(0.9310)
QARCH	3.2396	0.3201	**0.7640**	QARCH	**1.3394**	0.2357	0.0997
	(1.0000)	(1.0000)	(1.0000)		(1.0000)	(1.0000)	(1.0000)
**West Java**				**West Java**			
QAR	**0.4397**	0.0165	0.0620	QAR	**0.1693**	**0.0597**	**0.0153**
	(0.9692)	(0.7059)	(0.9310)		(0.9538)	(0.9412)	(0.9828)
QARCH	2.0909	**0.1391**	**0.2985**	QARCH	1.0961	0.2069	0.0700
	(1.0000)	(0.9804)	(0.9828)		(1.0000)	(1.0000)	(1.0000)

**Table 6:** Value-at-Risk forecast of Covid-19 risks in the period of pre-, during and post-lockdown-like policy (PSBB) in Province of South Sulawesi at 0.95 level of confidence and the corresponding coverage probability (in parentheses).

	$R_{t}$				$CFR_{t}$		
	Pre-PSBB	PSBB	Post-PSBB		Pre-PSBB	PSBB	Post-PSBB
QAR	0.2573	**0.1035**	0.0208	QAR	0.1131	0.0507	0.0323
	(0.8571)	(0.9655)	(0.4783)		(0.7778)	(0.1379)	(0.3152)
QARCH	**1.0581**	0.3561	**0.0949**	QARCH	**0.2109**	**0.0562**	**0.0370**
	(0.9714)	(1.0000)	(1.0000)		(0.8333)	(0.2414)	(0.7174)

	$RA_{t}$				$RCFR_{t}$		
	Pre-PSBB	PSBB	Post-PSBB		Pre-PSBB	PSBB	Post-PSBB

QAR	0.4098	**0.0970**	**0.0655**	QAR	0.2434	0.0283	**0.0541**
	(0.8710)	(0.9310)	(0.9022)		(0.8857)	(0.8621)	(0.9565)
QARCH	**1.2178**	0.2631	0.3249	QARCH	**0.9474**	**0.1521**	0.2366
	(0.9677)	(1.0000)	(1.0000)		(1.0000)	(1.0000)	(1.0000)

**Table 7:** Value-at-Risk forecast of Covid-19 risks in the period of pre-, during and post-lockdown-like policy (PSDD) in City of Jayapura and Province of Papua at 0.95 level of confidence and the corresponding coverage probability (in parentheses).

$R_{t}$				$CFR_{t}$			
	Pre-PSDD	PSDD	Post-PSDD		Pre-PSDD	PSDD	Post-PSDD
**Jayapura**				**Jayapura**			
QAR	0.1265	**0.1589**		QAR	**0.1531**	0.0154	
	(0.8889)	(0.9439)			(0.9444)	(0.6262)	
QARCH	**0.6107**	0.6496		QARCH	0.2778	**0.0241**	
	(1.0000)	(1.0000)			(1.0000)	(0.7944)	
**Papua**				**Papua**			
QAR	**0.6911**	0.0329	0.0161	QAR	**0.0841**	0.0113	0.0116
	(0.9687)	(0.6667)	(0.8947)		(0.9375)	(0.6961)	(0.8421)
QARCH	1.9496	**0.3510**	**0.0531**	QARCH	0.1836	**0.0146**	**0.0132**
	(1.0000)	(1.0000)	(1.0000)		(1.0000)	(0.7255)	(1.0000)
$RA_{t}$				$RCFR_{t}$			
	Pre-PSDD	PSDD	Post-PSDD		Pre-PSDD	PSDD	Post-PSDD

**Jayapura**				**Jayapura**			
QAR	0.1281	**0.2067**		QAR	0.0077	**0.0840**	
	(0.8889)	(0.9159)			(0.8750)	(0.9159)	
QARCH	**0.6192**	1.2428		QARCH	**0.2348**	0.5407	
	(0.9444)	(1.0000)			(0.8750)	(1.0000)	
**Papua**				**Papua**			
QAR	**0.4854**	0.0791	**0.0284**	QAR	**0.4822**	**0.0721**	**0.2674**
	(0.9063)	(0.8725)	(0.9474)		(0.9500)	(0.9608)	(1.0000)
QARCH	1.6193	**0.3173**	0.1920	QARCH	2.4132	0.3571	0.4261
	(1.0000)	(1.0000)	(1.0000)		(1.0000)	(0.9902)	(1.0000)

**Table 8:** Value-at-Risk forecast of Covid-19 risks in City of Medan, Province of North Sumatera, City of Samarinda and Province of East Kalimantan at 0.95 level of confidence and the corresponding coverage probability (in parentheses).

	Medan		North Sumatera		Samarinda		East Kalimantan	
	QAR	QARCH	QAR	QARCH	QAR	QARCH	QAR	QARCH
$R_{t}$	0.0714	**0.3194**	0.0961	0.3633	**0.1571**	0.4454	**0.1197**	0.3425
	(0.8446)	(0.9932)	(0.9177)	(0.9873)	(0.9286)	(1.0000)	(0.9317)	(0.9938)
$CFR_{t}$	0.0530	**0.0737**	0.0463	**0.0486**	0.0381	**0.0538**	0.0353	**0.0424**
	(0.2770)	(0.4864)	(0.0886)	(0.1203)	(0.8440)	(0.9858)	(0.9259)	(0.9691)
$RA_{t}$	**0.1437**	0.4977	0.1382	**0.5372**	**0.3016**	1.3906	**0.2247**	0.6725
	(0.9324)	(0.9932	(0.9114)	(0.9873)	(0.9357)	(1.0000)	(0.9752)	(1.0000)
$RCFR_{t}$	**0.0556**	0.2855	**0.1255**	0.5099	**0.1373**	0.7554	**0.2425**	0.7755
	(0.9456)	(1.0000)	(0.9682)	(0.9937)	(0.9615)	(1.0000)	(0.9653)	(1.0000)

## CRediT Author Statement

**Khreshna Syuhada:** Conceptualization, methodology, validation, formal analysis, writing original draft, writing review & editing, supervision; **Aqilah Wibisono:** Methodology, data curation, writing original draft, visualization; **Arief Hakim:** Methodology, validation, formal analysis, writing review & editing; **Fida Addini:** Methodology, writing review & editing.

## Declaration of Competing Interest

The authors declare that they have no known competing financial interests or personal relationships which have or could be perceived to have influenced the work reported in this article.
